# Parametric Bayesian modelling of tuberculosis mortality determinants and facility level heterogeneity effect using Gamma and Gaussian shared frailty techniques

**DOI:** 10.1186/s12879-026-12843-4

**Published:** 2026-03-11

**Authors:** Jacques L. Tamuzi, Isaac Fwemba, Veranyuy D. Ngah, Motlatsi Rangoanana, Llang Maama, Sele Maphalale, Mabatho Molete, Retselisitsoe Ratikoane, Modupe Ogunrombi, Olawande Daramola, Bonheur Dounebaine, Sara Adel Abbas, Peter S. Nyasulu

**Affiliations:** 1 Division of Epidemiology and Biostatistics, Faculty of Medicine, and Health Sciences, Stellenbosch University, Cape Town, South Africa; 2 Department of Epidemiology and Biostatistics, School of Public Health, University of Zambia, Lusaka, Zambia; 3 Disease Control Directorate, Ministry of Health Lesotho, National Tuberculosis Program, Ministry of Health, Maseru, Lesotho; 4 District Health Management Team, Butha Buthe, Ministry of Health, Lesotho National Tuberculosis Program, Ministry of Health, Maseru, Lesotho; 5 Department of Clinical Pharmacology, Sefako Makgatho Health Sciences University, Pretoria, Gauteng South Africa; 6 Department of Informatics, Faculty of Engineering, Built Environment & IT, University of Pretoria, Pretoria, Gauteng South Africa; 7 Africa CDC, Public Health Workforce Division, Addis Ababa, Ethiopia; 8 Central Agency for Public Mobilization and Statistics, Cairo, Egypt; 9 Division of Epidemiology & Biostatistics, Faculty of Health Sciences, School of Public Health, University of the Witwatersrand, Johannesburg, Gauteng South Africa; 10 Department of Clinical Pharmacology and Therapeutics, School of Medicine, Sefako Makgatho Health Sciences University, Pretoria, Gauteng South Africa

**Keywords:** Heterogeneity, TB mortality, Bayesian regression models

## Abstract

**Background:**

In a normal regression analysis for determinants of tuberculosis (TB) outcomes, assumptions that the sample is homogenous are made. However, the model does not account for the overall effect of unobserved or unmeasured covariates. Frailty may exacerbate the effects of TB on patients and raise their risk of death in Lesotho. This study aims to quantify the amount of heterogeneity that exists at the facility level, and to ascertain the determinants of TB mortality across all the catchment areas in Lesotho.

**Methods:**

This was a retrospective cohort of patients on TB treatment registered from January 2015 to December 2020 at twelve (12) health care facilities in the district of Butha Buthe, Lesotho. Data were collected from patient’s medical records and analyzed using R and Integrated Nested Laplace Approximation (INLA). Descriptive statistics were presented using frequency tables. Differences between binary outcomes were analyzed using Pearson’s *X*^*2*^ test. Mixed effect model with five Bayesian regression models of varying distributions were used to assess heterogeneity at facility level. We reported results using the log-logistic regression model with a Gamma frailty term. Kaplan-Meier curves were used to demonstrate time-to-death events.

**Results:**

The total number of patients included in the analysis were 1729 of which 70% were males, and about half of them were employed (54.2%). Being over 60 years (HR: 0.14, Cl: 0.02–0.94) 20–59 years (HR: 0.07, Cl: 0.01–0.41), and TB category 2 (HR 0.27, Cl 0.09–0.80) decreased the risk of dying. Miners had an increased risk of dying from TB (HR:4.13, Cl: 1.05 - 16.43). Assessing heterogeneity by varying the frailty distribution revealed interesting results. For instance, specifying frailty variance structure to follow gamma distribution resulted in estimating minimal 1.01 heterogeneity between catchment areas. The heterogeneity estimated by specifying the Gaussian distribution for the frailty term resulted in nearly 4.5 times more likely to have increased the risk of dying.

**Conclusion:**

The results from both Gamma and Gaussian demonstrate that heterogeneity affected significance of the determinants for TB mortality. The results showed that facility level was significantly likely to increase the risk of dying, indicating differences between catchment areas.

## Highlights

1. Reports of being employed as a miner associated with higher TB mortality is worrying. This finding may help authorities in Lesotho and the African region to design health strategies that can target miners and those living within the mining catchment areas.
2. The use of community health workers and close relatives reduced the risk of dying among TB patients. This is a key factor that can be considered in designing effective TB interventions in Lesotho. Ensuring that each patient is assigned a community health worker may reduce mortality.
3. The risk of death was significantly higher in treatment phase 2 among patients with pulmonary TB compared to patients in treatment phase 1 and among those with extra pulmonary TB.

**Strength of the study**

4. The study is based on mixed effect models with varying distribution for the frailty parameter. Selecting baseline hazard distribution is based on how the Lesotho data fitted the model and not based on historical practice that is not motivated by current data.
5. Presence of heterogeneity at a facility level means that interventions meant to improve TB treatment outcomes must be taken into consideration seriously.

## Background

Tuberculosis (TB) is one of the main leading causes of mortality worldwide from a single infectious disease [1]. The highest burden of the disease is found in sub-Saharan Africa with 25% of worldwide incident cases in 2020 as reported by the World Health Organization [1, 2].

TB control is a serious public health priority in many low- and middle- income countries (LMIC) as death rates among patients on TB treatment is still increasing globally [3, 4]. Several factors affecting survival of patients are missed in the TB treatment programs. Many studies assessing risk factors for survival of TB patients use survival statistical analysis which does not take into consideration the natural variability of the different environments among the patients [5].

In a normal regression model of tuberculosis treatment outcome in a specific population, the assumption that the sample is homogenous is made. Not only is this assumption unrealistic, but it is also impractical. This is because there are certain demographic differences among patients which researchers are unaware of [6, 7]. In most studies, the burden of obtaining all the necessary explanatory variables causes limitations due to important covariates being missed. This results in unwanted heterogeneity or clustering effect in the study [3, 4].

Determining community level (frailty parameter) clustering effect is not new in biomedical research, but requires that the survival times for participants be independent and identically distributed [8]. This assumption has been violated in practice, especially when data being analyzed involves participants that are connected on account of their relation within a family [9], or by sharing facility and environment factors (8–14). Similarly, other studies on TB outcomes, have shown that cluster levels factors (facility level) may have significant effects on mortality [10]. Regrettably, facility effects are typically not accounted for in studies assessing determinants of health outcomes such as TB. Patients who are already frail are more likely to die from TB, and frailty can have a substantial impact on TB patients’ overall survival. Frailty, defined as diminished physical function and greater susceptibility to comorbidities, can exacerbate the effects of TB on patients and raise their risk of death. The use of frailty models have only been used recently in TB mortality studies [11, 12].

Determinants of this nature represent the cumulative effect of unobserved or unmeasured covariates that may reflect impacts of environmental and socio-cultural factors. Various statistical methods are used to evaluate the size of the effect of these unobserved factors which have been known to act either multiplicatively or additively on the baseline hazards [8, 13]. Hougaard [14], and Klein [15], modelled the dependence of the covariate structure via frailty model of an assumed parametric distribution. Woya and Jabir et al., modelled the baseline hazard function using a Gamma shared frailty model and concluded that there was no heterogeneity for death in patients treated in different hospitals [12]. This study found that covariates such weight, age, extra pulmonary type of TB, and HIV status of TB patients were significant risk factors associated with death status among TB patients.

In practice modelling of frailty parameter decision has been done on the basis of traceability of the frailty function and availability of appropriate software [16] to implement these models and, not necessarily on their scientific merit. Lack of scientific evidence to support such decisions has affected appropriate inference needed to correctly estimate variables needed to inform targeted TB interventions. This study aimed i) to examine how well a proposed distribution fits the data before any inference can be drawn, ii) to quantify the amount of heterogeneity existing at community level in Butha Buthe, Lesotho, which may bias mean estimates with their corresponding confidence intervals and consequently the credible intervals and lastly to iii) to identify determinants of TB mortality across all the catchment areas in Butha Buthe Lesotho.

## Methods

### Data sources and variables

This was a retrospective study conducted in ten primary care facilities and two secondary level hospitals in Butha-Buthe district. TB data were gathered from records of adult TB patients registered in health care facilities from 12 regions (including Butha-Buthe Government hospital (BBGH), Seboche Hospital, Boiketsiso, Linakeng, Makhunoane, Motete, Muela, Ngoajane, Rampai, St Paul, St Peters, and Tsime clinics). A trained research coordinator supervised the process of data collection and ensured effective data quality controls. The current data contained parameters on 1729 TB patients. These patients were diagnosed with TB between the period of January 2015 and December 2020. Data collected included patients’ demographics (sex and age of the participant), TB outcomes, facility, TB treatment category, medication start date, medication completion date, phase of treatment, treatment contact group, and occupation.

To ensure data quality, we conducted a verification exercise by going through each facility’s records to ensure all erroneous entries were corrected or removed. We also verified TB outcomes (cure, death, lost to follow-up and transfer) and performed data consolidation. The outcome variable was defined as survival time in days of patients who were diagnosed with TB. Those who died because of TB were patients classified as having had the event and assigned to the number ‘1’. Patients who either dropped out of the study or transferred out were censored using mechanism as illustrated in Eq. (8) and were assigned the number ‘0’. Frailties are random effects models that are used to account for association or unobserved heterogeneity of clustered survival data such as the one from Lesotho [17].

### Statistical approach

Bayesian methodology was used to fit several parametric regression models. Fifteen models were specified for this work. The first five were Bayesian regression models (log-logistic, log-normal, exponential, Weibull and Gompertz) specified and fitted with the assumption that heterogeneity (frailty) is homogenous across the catchment areas. This assumption means that the five models were fitted using fixed effects model approach.

Following the assumption of proportional hazards, we expressed the time to failure t as shown:

1 $${h_i}\left( t \right) = {h_0}\left( t \right)exp\left( {{\eta _i}{\rm{ }}} \right),t{\rm{ }} > {\rm{ }}0,$$

hence the baseline hazard is represented by ${h_0}\left( \cdot \right)$, and the exposures by ${\eta _i}$,

In this analysis, we made assumptions that the TB data from Lesotho follows the right censoring mechanism, where patients who died from TB have experienced the event while all others (such as lost to follow-up, dropouts, and transfer outs) were censored (have not had the event of interest).

### Exponential distribution

We assumed that the hazard function is constant over time. We thus assumed that the age of a patient had no impact on their future survival. Survival time *t* follows an exponential distribution with parameter λ and corresponding pdf as shown below. We assumed that survival time, t follows a Gompertz distribution with parameter λ and corresponding pdf as shown below:

2 $$f(x) = \lambda {e^{ - \lambda t}},t > \lambda > 0$$

Where $$h(t) = \lambda $$

### Gompertz distribution

We assumed that survival time with probability density function of the Gompertz distribution can be expressed as shown below:

3 $$f\left( x \right) = \lambda {e^{\theta t}}\exp ({\lambda \over \theta }\left( {1 - {e^{\theta t}}} \right),t > 0,\lambda > 0$$

$$h(t) = \lambda {e^{\theta t}}$$ and $$\exp [(\lambda /\theta )(1 - {e^{\theta t}})]$$

### Weibull distribution

The Weibull distribution is suitable for modeling data with monotone hazard rates that increase or decrease exponentially over time. For Weibull regression, λ is the scale parameter and γ is a shape parameter, and its probability distribution function is as shown below:

4 $$f(t) = \lambda \gamma {t^{\gamma - 1}}{e^{ - \lambda t\gamma }},\lambda ,\gamma > 0,$$

Expressing the latent field x, in terms of the predictor ${\eta _i}$, the standard Weibull regression is expressed as ${h_i} = {\lambda _t}{t^{\lambda - {\rm{ }}1}}exp({\eta _i}{\rm{) }}$

$${\rm{s(t)}} = {\rm{ }}{e^{ - \lambda t\gamma }}$$ and $${\rm{h(t)}} = \lambda \gamma {t^{\gamma - 1}}.{\rm{ }}$$

### The log-logistic distribution

A limitation of the Weibull hazard is its monotonic properties as a function of time. However, we considered circumstances where the direction of the hazard function changes. In such situation, the model of choice is the log-logistic:

$$f(t) = {{{e^\theta }k{t^{\gamma - 1}}} \over {{{(1 + {e^\theta }{t^k})}^2}}}$$

5 $$h(t) = {{{e^\theta }k{t^{k - 1}}} \over {{{(1 + {e^\theta }{t^k})}^2}}}$$

For $$0 \le t < \propto $$ and $$k > 0$$ $$s(t) = {1 \over {(1{\rm{ }} + {\rm{ }}{e^\theta }{t^k})}}$$

### The log-normal distribution

We specified the log-normal distribution to model TB survival data from Lesotho. It is thus assumed that a variable that is random has a distribution that is log-normal with parameters $\mu$ and δ^2^ log T, with a mean parameter $\mu$ and variance δ^2^. Using this information, the density function of T can be defined as follows:

6 $$f\left( t \right) = {1 \over {\delta \sqrt {2\pi } }}{t^t}\exp ({{ - {{(\log t - u)}^2}} \over {2{\delta ^2}}},0 \le t\left\langle {\infty ,\delta } \right\rangle 0.$$

The survival function of the log-normal distribution is:

$$s(t) = 1 - \phi (\log t - \mu )/\delta $$

Where $\phi (.)$is the standard normal distribution function given by

$$\phi (z) = (1){(2\pi )^{1/2}}\int\limits_{ - \infty }^z {\exp ( - {u^2}/2)du} $$ and $$h(t) = f(t)/S(t)$$

### Approach for determining catchment level heterogeneity

To determine the catchment level heterogeneity, we varied the assumptions to model and analyze the time to death of TB patients by considering frailty survival models. In a shared frailty model, we assume that the lifetimes of a group of observations in the same cluster share the same level of frailty [8, 12, 18, 19]. Parametric models are more advantageous in that they fit the data better than semi parametric, especially if the shape parameter of the hazard function is known.

Ten (10) Bayesian frailty regression models were fitted in addition to the first five fixed effect models fitted earlier as explained (Weibull, exponential, log-logistic, lognormal, and Gompertz). The survival mixed effects (frailty) models fitted included five Gamma frailty specified models and five Gaussian models with a shared frailty term. Inclusion of the frailty term was meant to capture any significant unobserved effect (presence of heterogeneity) that may be present at catchment area level.

## Frailty model

Assuming we have m catchment areas with each catchment area $i$ having ${n_i}$ observations with total sample size $\sum\limits_{n = 1}^m {{n_i} = } n$. Let the observed failure time with a right censoring scheme for the ${j^{th}}\left( {j = 1,{\rm{ }}2,{\rm{ }}.{\rm{ }}.{\rm{ }}.{\rm{ }},{\rm{ }}{n_i}} \right)$, observation in the ${i^{th}}$ catchment area, and ${c_{ij}}$ be the censoring time; then failure time $j_{ij}^*$ and censoring time ${c_{ij}}$, assumed to be independently distributed random variables [18, 20], are defined as ${t_{ij}} = min({c_{ij}},{\rm{ }}t_{ij}^*)$. In addition, we consider the random variable ${v_i}$ be the frailty effect of the ${i^{th}}$ catchment areas with known distribution function ${f_v}({v_i})$. Conditional on the frailty variable ${v_i}$ and covariates ${x_{ij}}$, the survival function ${s_{ij}}(.|{x_{ij,}}{v_i})$ at time $t$ of the ${j^{th}}$ observation of catchment area $i$, is given by [17, 19, 21] as shown below;

7 $${s_{ij}}(t|{x_{ij}},{v_i}) = \exp \{ - {\Lambda _0}(t){v_i}{e^{x_{ij}^,\beta }}\} $$

the cumulative baseline hazard function (${\Lambda _0}\left( t \right)$), which was specified during survival data generating mechanism, and $\beta$ represents the unknown regression parameters. The failure timed can be determined with the invertible ${\Lambda _0}\left( t \right)$ as follows,

8 $$t_{ij}^* = \Lambda _0^{ - 1}\{ - \ln ({U_{ij}}{e^{ - {{x_{ij}^;} \over {{v_i}}}}}\} $$

where, ${U_{ij}} \sim U\left( {0,{\rm{ }}1} \right)$and $t_{ij}^*$ is the failure time of member $j$ of catchment $i$. Then the perceived censoring measure ${\delta _{ij}}$ is 1 if ${c_{ij}} > t_{ij}^*$, and 0 otherwise. Taking into account frailty term ${\nu _i}$ and covariate ${x_{ij}}$, the shared frailty model at time $t$ of the ${j^{th}}$ observation of catchment $i$, can be specified as shown in the model below;

9 $${\lambda _{ij}}\left( {t|{x_{ij}},{\nu _i}} \right) = {\nu _i}{\lambda _0}\left( t \right)exp\{ x_{ij}^\prime\beta \} ,$$

where ${\lambda _0}\left( . \right)$is the baseline hazard function, while vector of the regression parameters is represented by $\beta$. We note that,if ${n_i}{\rm{ }} = {\rm{ }}1$ for $i{\rm{ }} = {\rm{ }}1,{\rm{ }}2,{\rm{ }}.{\rm{ }}.{\rm{ }}.{\rm{ }},{\rm{ }}m,$ the survival function in expression (1) and the hazard function in expression (2) changes to a proportional hazard model [22]. The frailty effects ${\nu _{i{\rm{ }}}},{\rm{ }}i{\rm{ }} = {\rm{ }}1,{\rm{ }}.{\rm{ }}.{\rm{ }}.{\rm{ }},{\rm{ }}m$ are presumed to be independent and homogeneously distributed random variables that can assume distribution functions such as log-normal, positive stable distribution, Gamma and inverse Gaussian distributions [17, 18].

Due to their computational convenience, the Gamma and the inverse Gaussian distributions were used as the frailty distributions for this study. The Gaussian frailty model was chosen because it can model dependencies both between and within groups, allowing data to be shared while capturing different group-specific distributions [23]. While the Gamma frailty model was chosen based on the fact that the parameters associated to the shape of the Gamma distribution enables it to conform to many distributions, such as exponential and chi-squared, offering a more refined instrument for discerning factors influencing TB treatment outcomes [24].

### Gamma frailty model

Gamma density function is defined by the two-parameter frailty expression ${v_i}$ which has a shape parameter $k$ with scale parameter $\lambda$ formulated as shown below

10 $$f\nu \left( {{\nu _i}} \right){\rm{ }} = {{{k^\lambda }\nu _i^{\lambda - 1}{e^{ - k\nu i}}{\rm{ }}} \over {\Gamma \left( \lambda \right){\rm{ }}}};\lambda > 0,{\rm{ }}k > 0$$

While expression 11 shows the Laplace transformed version with the density [8] form shown below:

11 $$L\left( s \right){\rm{ }} = {\rm{ }}\int_0^\infty {exp\left( { - {\nu _i}s} \right){f_\nu }\left( {{\nu _i}} \right)d{\nu _i}} = {\rm{ }}{k^\lambda }{\left( {s{\rm{ }} + {\rm{ }}k} \right)^{ - \lambda }}$$

The solutions of the primary and secondary partial derivatives of the Laplace function L(.)in regards to $s$ equal to 0, give the mean ${v_i},{u_v} = E({v_i}) = {k \over \lambda }$ and variance of the frailty term $\sigma _v^{^2} = {\rm{ }}Var\left( {{\nu _i}} \right) = {\rm{ }}{k \over {{\lambda ^2}}}{\rm{ }}$, respectively. We set mean ${\mu _\nu }$ of the frailty term equal to 1 and the variance of the frailty term $\sigma _\nu ^2{\rm{ }}$ becomes $\theta = {1 \over \lambda }{\rm{ }}$, in order to attain model identifiability. The Gamma frailty model for individual $j$ in facility catchment area $i$ is therefore calculated as:

12 $$\lambda \left( {t|{x_{ij}},{\nu _i}} \right) = {\nu _i}{\lambda _0}\left( t \right)exp\{ {x_{ij}}\beta \} = {\nu _i}\lambda \left( {{t_{ij}}} \right)$$

### Inverse gaussian frailty model

Like the Gamma frailty model, simple closed-form expressions exist for the unconditional survival and hazard functions. We can express the probability density function of an inverse Gaussian distributed frailty random variable ${\nu _i}$ with parameters µ > 0 and $\alpha {\rm{ }} > {\rm{ }}0$ as expressed in [8, 22] as shown below,

13 $$f\nu \left( {{\nu _i}} \right) = {\rm{ }}{{{\alpha ^{1/2}}} \over {\surd 2\pi }}v_i^{ - 3/2}exp\{ - {\rm{ }}{\alpha \over {2{\nu _i}{\mu ^2}}}{\left( {{\nu _i} - \mu } \right)^2},$$

Laplace transform of this function is configured as,

14 $$L\left( s \right){\rm{ }} = {\rm{ }}exp{\rm{ }}\left\{ {{\alpha \over \mu } - {{({{({\alpha \over \mu })}^2} - 2\alpha s)}^{1/2}}} \right\};s \ge {\rm{ }}0.$$

### Construction of the likelihood function (Models with and without frailty terms)

The proportional hazard model specifies that the hazard at some time t for individual with covariate ×can be expressed as

15 $$h(t|x) = {h_0}(t)\exp ({X^\prime}\beta )$$

Where baseline hazards function is represented by${h_0}(t)$, X representing the vector of covariates, $\beta$ denotes the regression coefficients and ${S_0}({t_i})$ the survival function. The likelihood function $L(D|{h_0}({t_i}),\beta )$ was expressed in the form of a right censored data (for the TB mortality) on n number of subjects as given below:

16 $$L(D|{h_0}({t_i}),\beta ) = \Pi _{i = 1}^n{\left\{ {{h_0}({t_i})\exp ({X^\prime}\beta )} \right\}^{{\delta _i}}}({s_0}{({t_i})^{\exp ({X_i}\beta )}})$$

We used Bayesian estimation procedure using a deterministic (INLA) technique to estimate the parameters involved in these models. We fitted 15 models and compared their WAIC values to select one best model. Use of INLA has been common especially in fitting complex mixed effect models such as involving frailty terms. Using the INLA approach, we specified a shared frailty model which implies that similar observations within a group have similar characteristics or frailty, but these frailties differ between communities as earlier stated in the manuscript. We assumed that the survival times for say the ${i^{th}}$ TB patient (i = 1,n) in the ${j^{th}}$ group(j = 1 … .m) is denoted by ${t_{ij}}$with an unobserved frailty parameter given as ${u_i}$(for the ${j^{th}}$group). With this configuration, the hazard function for the proportional hazards model was given as

17 $$h({t_{ij}}|{X_{ij}},{u_j}) = {h_0}(t{}_{ij})\exp ({X_{ij}}\beta ){u_j}$$

Where ${u_1}.....{u_m}$represent the frailty and${h_0}(t)$, ${X_{ij}}$and $\beta$are the baseline hazards, vector of covariates and regression coefficients respectively. The ${u_j}$is assumed to be independent and identically distributed with mean 1 and variance$\theta$. The frailty distribution for each ${u_j}$is assumed to be independent gamma as presented below:

$${u_j} \sim \sim Gamma(\eta ,\eta ),j = 1....,m$$

Where $\eta$is the unknown variance of ${u_j}$. Using this information, we specify the distribution for the frailty as below:

$$X \sim \sim Gamma(a,b) \propto {x^{a - 1}}\exp ( - bx),$$for x > 0, a > 0, b > 0

### Posterior distribution

The posterior probability density function which summarizes our beliefs about a particular parameter is constructed using Bayesian theorem as

18 $$\pi (\theta |D) = {{\pi (\theta )L(D|\theta )} \over {\int_\Theta {\pi (\theta )L(D|\theta )d\theta } }}$$

Which can be summarized as

19 $$\pi (\theta |D) \propto \pi (\theta )L(D|\theta )$$

Therefore, we constructed our posterior distribution based on the likelihood function defined in terms of the frailty as shown below:

20 $$\pi ({h_{_0}}(t),\beta |D) \propto {\prod\nolimits_{i = 1}^{\tilde n} {\left\{ {{h_0}({t_{_i}})\exp ({X_i}\beta ){u_i}} \right\}} ^{{\delta _i}}}({s_0}{({t_i})^{\exp ({X_i}\beta )}})\pi (\beta )$$

### Prior information

We specified normal distribution with ${\mu _0} = 0$,variance $\sigma _0^2 = 100$ as priors for the regression coefficient ${\beta _i}$whose normal probability density function is captured as below;

21 $$f(x|{\mu _0},\sigma _0^2) = {1 \over {\sqrt {2\pi \sigma _0^2} }}{e^{ - {{{{(x - {\mu _0})}^2}} \over {2\sigma _0^2}}}}$$

For the frailty component, frailty parameters were analyzed via Bayesian framework with gamma distribution specified as appropriate prior distribution with mean being equal to 1 and variance of 1000. The selection of vague to weak priors was informed by literature and for us to be able to interpret the finding using Bayesian way. Other priors such as vague and flat priors ($\tau \sim\Gamma \left( {1,{\rm{ }}1} \right)$) were also assigned and used to check for sensitivity and robustness of the estimates.

### Test of proportionality under survival analysis and other model diagnostics

We used Schoenfeld residual approach to test for the proportional hazard’s assumption conditions and the graphical approach. The Schoenfeld test hypothesizes that some variables do not vary with time. This hypothesis implies that variables remain constant over the study period and therefore satisfied the proportionality assumption under the standard survival PH model. We stratified variables that did not satisfy this condition but were significant.

### Model diagnostic and assessment

Model evaluation in INLA (Integrated Nested Laplace Approximation) typically focuses on comparing and assessing model fit, complexity, and predictive performance. On the other hand, assessing convergence in INLA involves evaluating the accuracy of the numerical approximation of the posterior marginal. A nested Laplace approximation is used to approximate the posterior distribution, and it’s crucial to ensure this approximation is accurate enough for reliable inference. Thus, we used several techniques to assess convergence (diagnostic) in INLA. These included the following:

**Looking out for warning messages:** Whilst fitting the models, we looked out for a warning message that INLA would flag out whenever there is a problem with hyper parameters prior specifications or the approximation itself. The models specified did not have any warning messages and as such we are comfortable that the estimation process was ok.

**Evaluating the Deviance Information Criteria and Watanable Akaike Information Criteria (WAIC):** The Bayesian measure analogues to the frequentist Akaike Information Criteria was also used to assess the model fit. Comparison of the models were carried out using the deviance information criteria (DIC) and the Watanabe Information Criteria (WAIC). The DIC is the Bayesian version of the frequentist AIC and BIC. It has two components, the goodness of fit represented by $\mathop D\limits^ - (\theta )$ and the model complexity term pD. This in effect makes DIC= $\mathop D\limits^ - (\theta )$ + pD. Smaller values of the DIC are preferable to larger values.

We also checked the Conditional Predictive Ordinate (CPO) and Probability Integral Transform (PIT) metrics. CPO Measures how well the model predicts each observation, using the leaving it out (leave-one-out cross-validation) validation criteria. We assessed the PIT for the log- log Gaussian and gamma frailty which showed to be uniformly distributed. In this model both the CPO and PIT were used to check calibration and individual predictive performance. Further, we also inspected the distribution of the posterior marginal. This was done by examining the shapes and distribution of the estimated parameters and hyper parameters from the model. All the quantities evaluated looked reasonable.

### Reporting estimates

For categorical measures frequencies and associated proportions were determined and compared using the Chi-square test. To determine differences in survival by sex, age category and catchment areas, Kaplan–Meier test was used.

Univariate and multivariate shared Gamma and Gaussian frailty models were fitted using integrated nested Laplace approximation (INLA), to determine factors associated with TB mortality. Estimates generated from INLA were exponentiated to obtain posterior summaries.

Hazard ratios were used to report measures of effect with their associated 95% credible intervals. A 95% confidence interval excluding one was determined as significant. Model fit was assessed using the deviance information criterion (DIC) and Watanabe Information Criteria (WAIC). All statistical analyses were performed using R for descriptive statistics, while inference component was implemented using deterministic Bayesian based approach known as INLA, which is a package implemented in R open-source software.

The leading performing model is the Log-logistic with or without the frailty term for both Gamma and Gaussian distribution, hence the strongest and decisive model is the log logistic model with a Gamma shared frailty distribution model. Thus, the concluding analysis and results interpretation were established on the logistic regression model with a Gamma frailty term. However, for the heterogeneity assessment, the Gaussian frailty model was preferred and found to be more stable and the frailty parameter value obtained was more realistic. Despite having different frailty variance structure, the two models gave similar values.

## Results

In Table 1, we provide a summary of the covariates included in the analysis. Evident disparities by catchment areas (facility names) were noted, with Seboche, and BBGH catchment areas having the highest number of TB patients accounting for 50%, and 22.9%, respectively. Most common disease characteristics observed in the studied catchment areas included: Phase 1 of treatment (64.6%) and having pulmonary TB (77.1%). Overall, 19.1% TB related deaths were reported, and 80.9% TB patients survived the TB episode. In this study nearly Eighty-nine percent (89.43%) of TB cases were category 1. Majority of the participants (70.1%) in the study were males compared to females (29.9%).

**Table 1:** Distribution of characteristics of the study participants

Variables	Frequency (%)
**Sex**	
Female	513 (29.7%)
Male	1213 (70.3%)
**Employment status**:	
Employed	935 (54.2%)
Unemployed	791 (45.8%)
**Age category**	
<20 Yrs.	69 (4.00%)
≥60 Yrs.	378 (21.9%)
20–59 Yrs.	1279 (74.1%)
**Occupation Category**:	
Employed non-mine worker	390 (22.6%)
Mine Worker	549 (31.8%)
Unemployed	787 (45.6%)
**TB Category:**	
Extra pulmonary TB	403 (23.3%)
Pulmonary TB	1323 (76.7%)
**Treatment contact group:**	
CHW	267 (15.5%)
Family Members	866 (50.2%)
Friend	152 (8.81%)
Unknown	441 (25.6%)
**Phase Of treatment:**	
Phase 1	1103 (63.9%)
Phase 2	623 (36.1%)
**TB category**	
CAT 1	1.607(89.43%)
CAT 2	161(8.96%)
CAT 3	20(1.11%)
CAT 4	9(0.50%)
**Healthcare Facilities**	
BBGH	380(22.02%)
Boiketsiso	77(4.46%)
Linakeng	45(2.61%)
Makhunoane	33(1.91%)
Motete	8(0.46%)
Muela Botha	24(1.39%)
Ngoajane	47(2.72%)
Rampai	24(1.39%)
Seboche	884(51.22%)
St Paul	89(5.16%)
St Peters	57(3.3%)
Tsima	58(3.36%)

## Comparison of survival curves

**Fig. 1:**
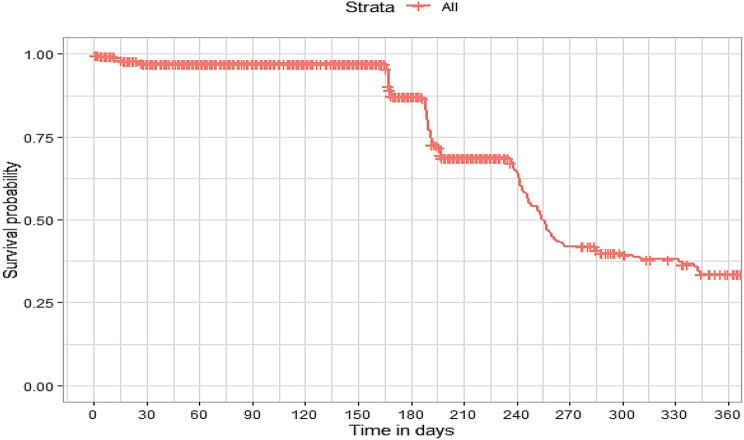
Overall survival

**Fig. 2:**
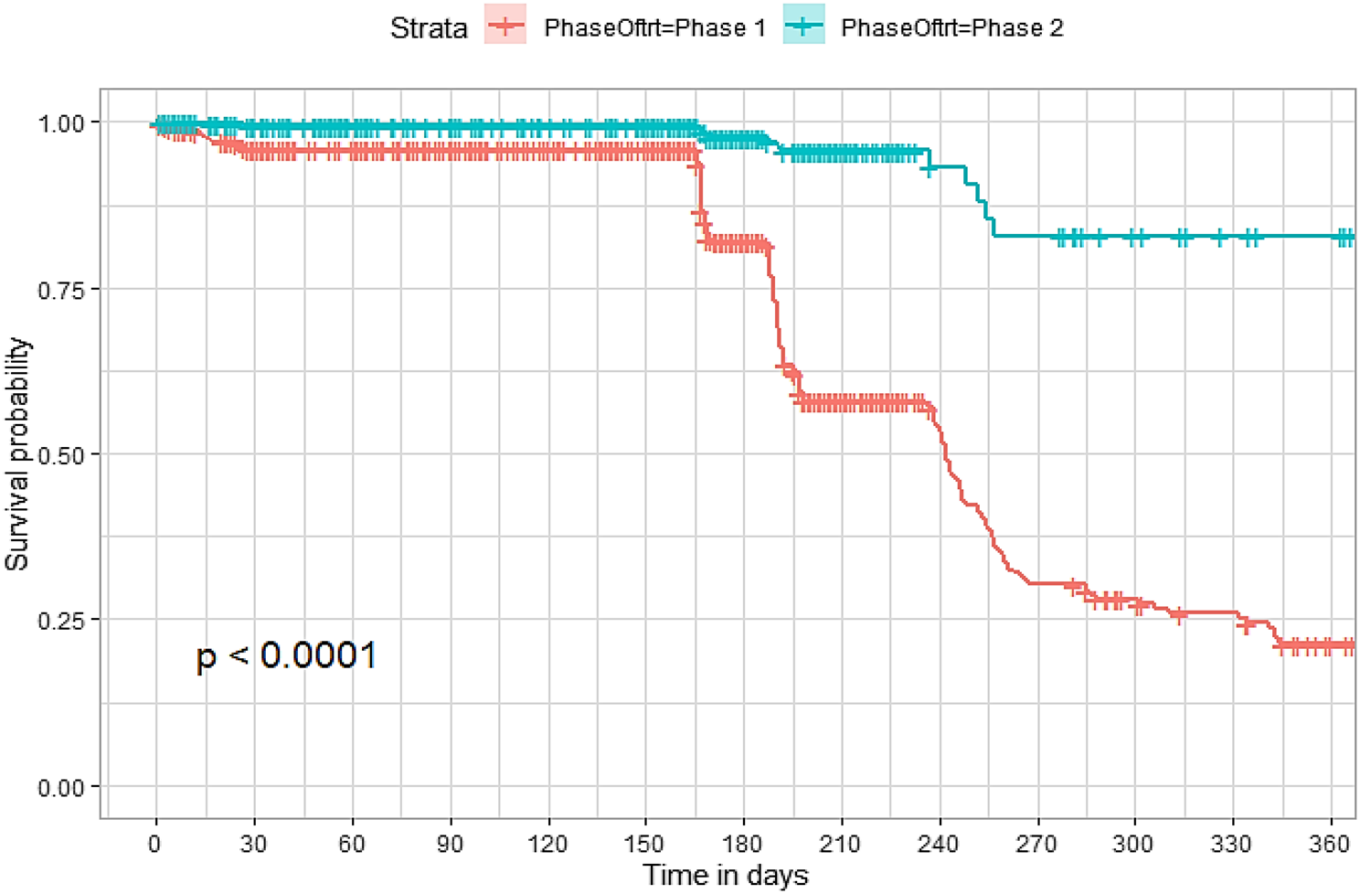
Survival curves for phase of treatment for the study participants

**Fig. 3:**
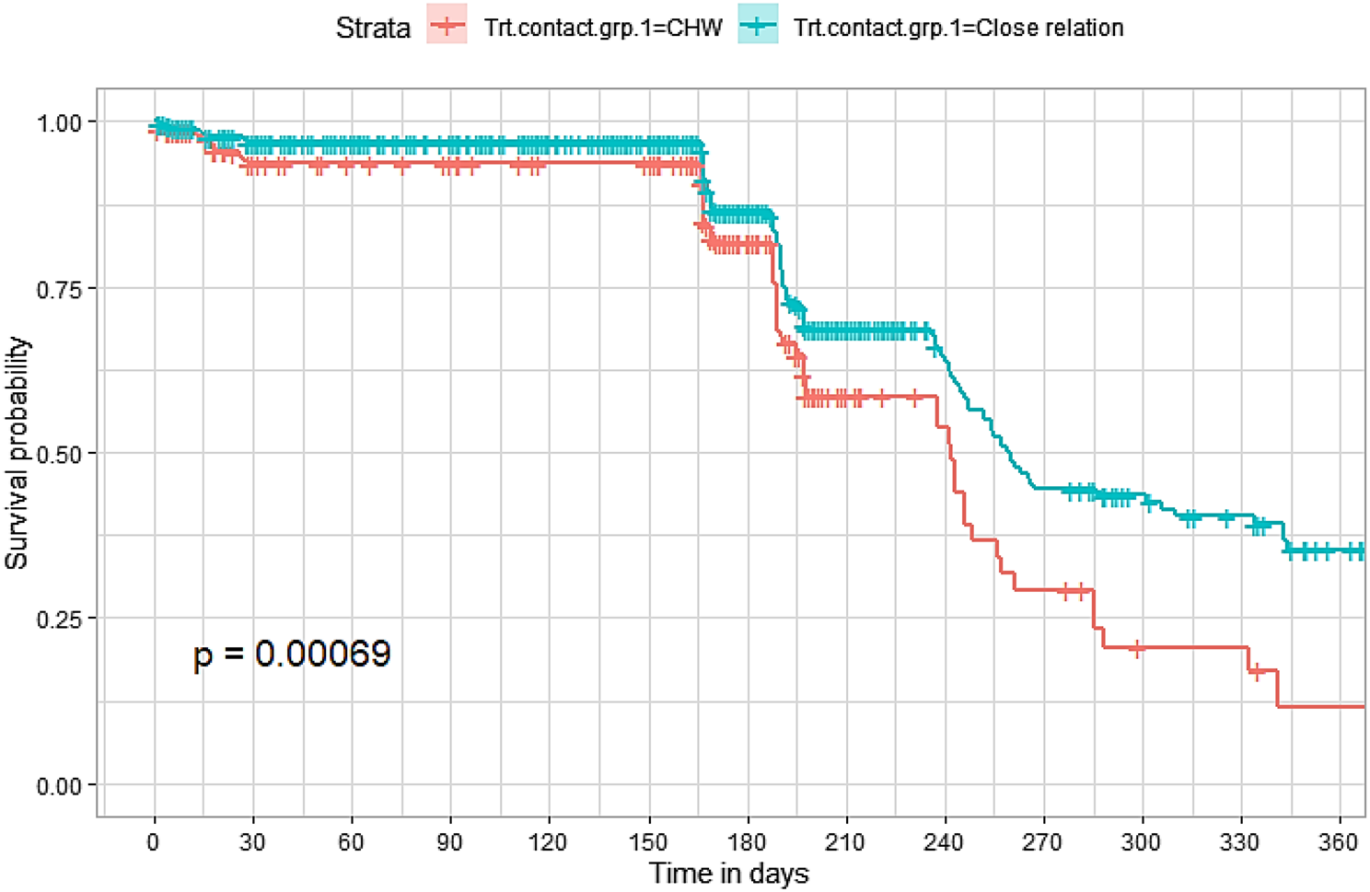
Survival curves for treatment contact for the study participants

**Fig. 4:**
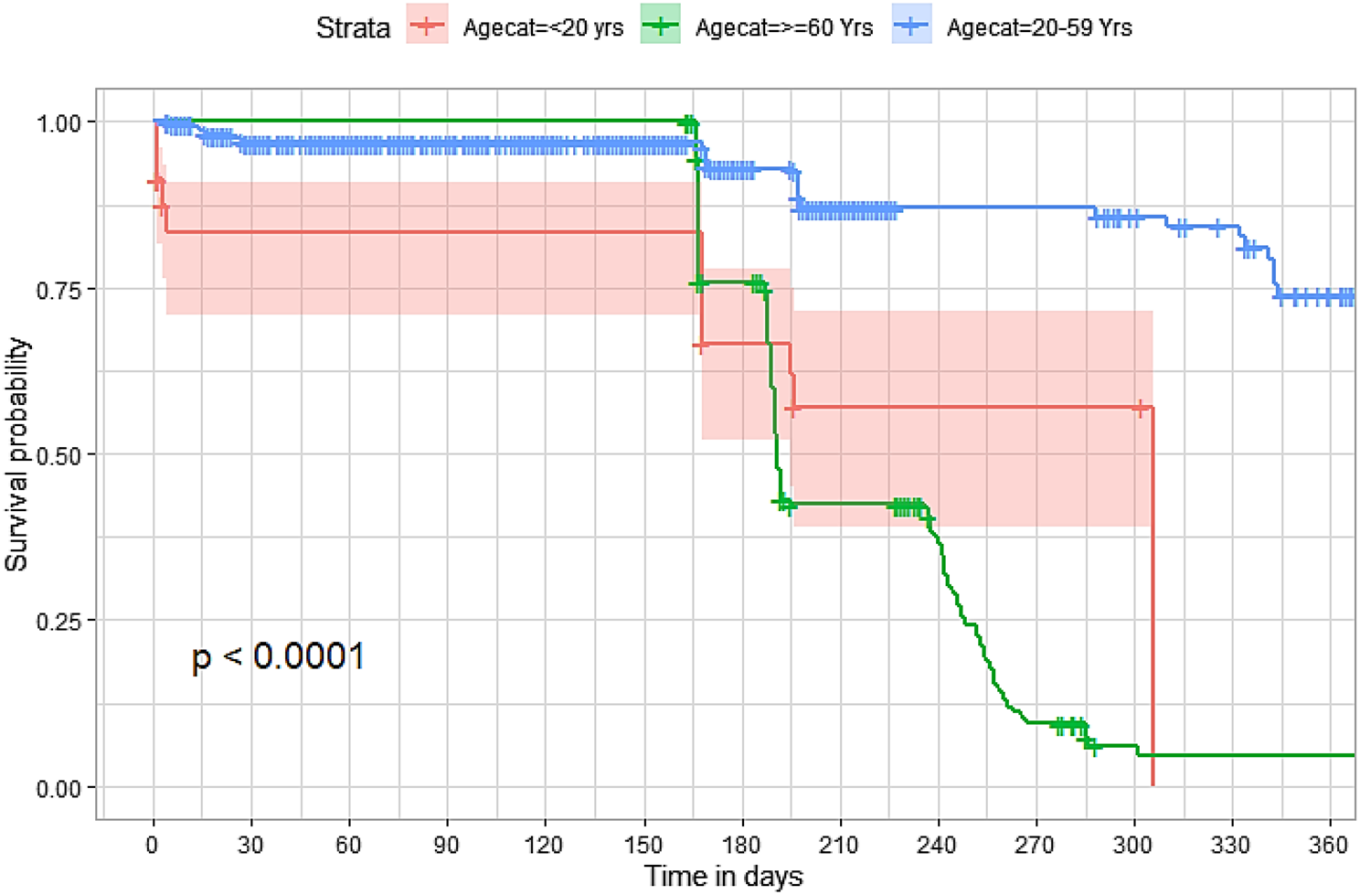
Survival curves for age category for the study participants

**Fig. 5:**
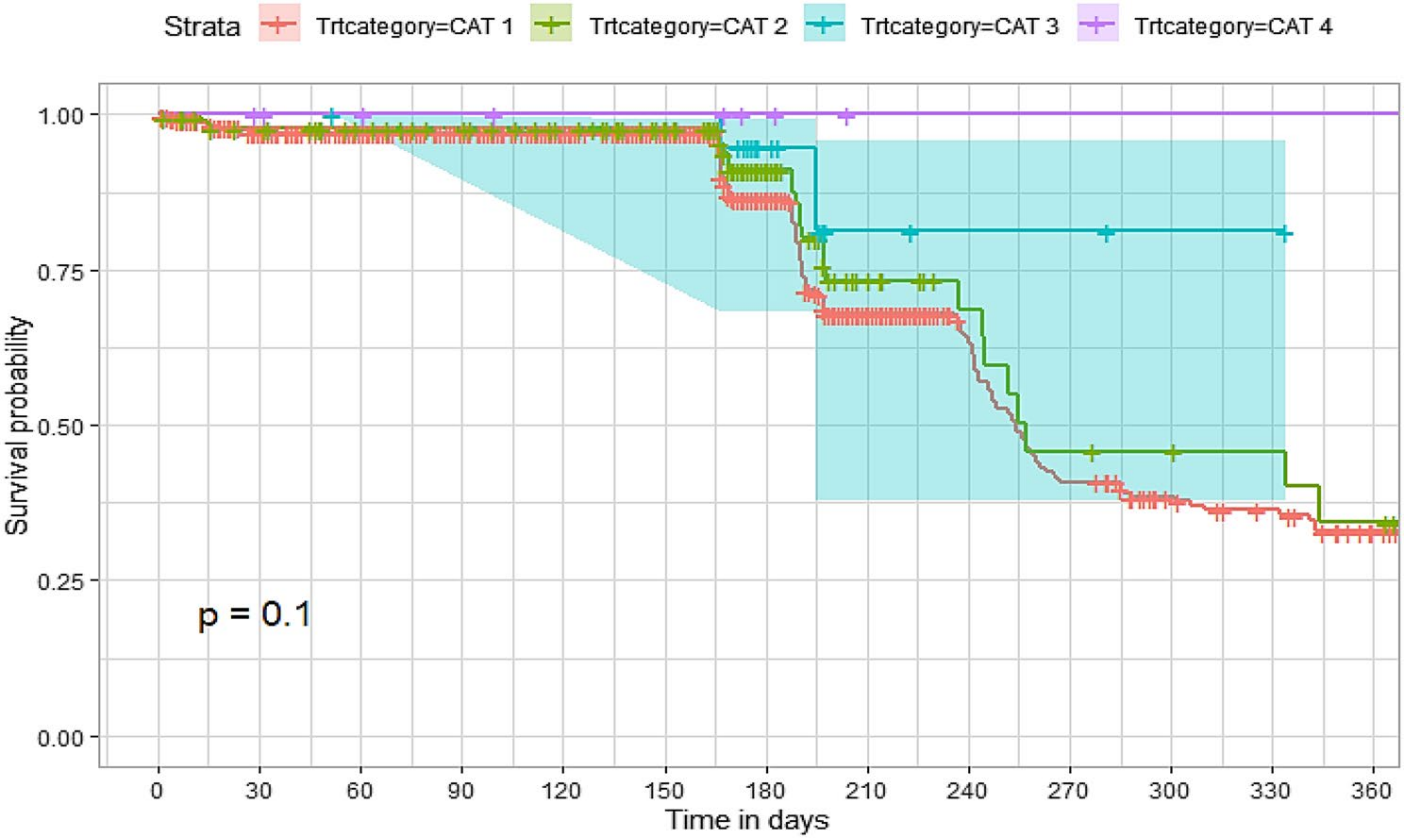
Survival curves for TB treatment category for the study participants

**Fig. 6:**
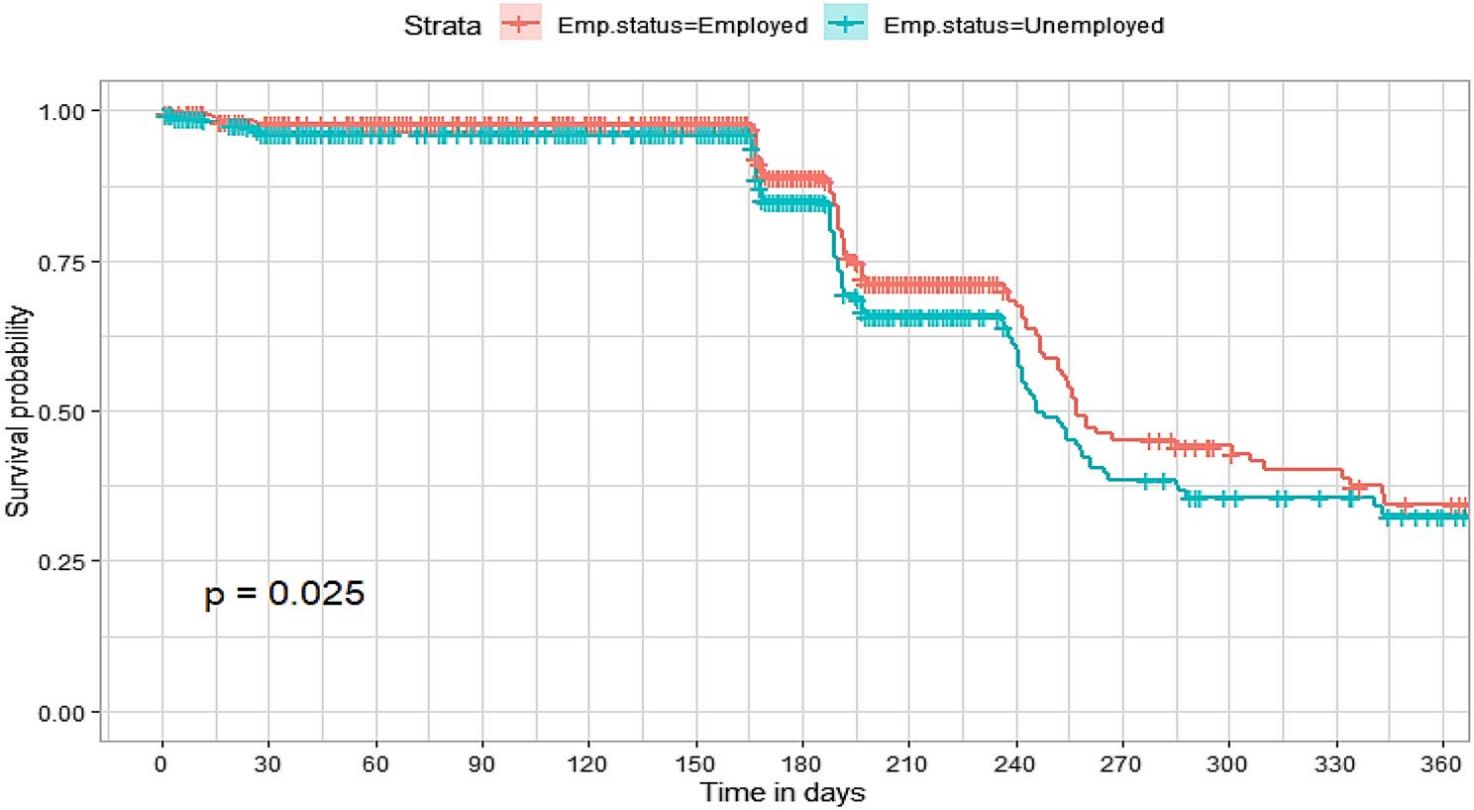
Survival curves for employment status for study participants

**Fig. 7:**
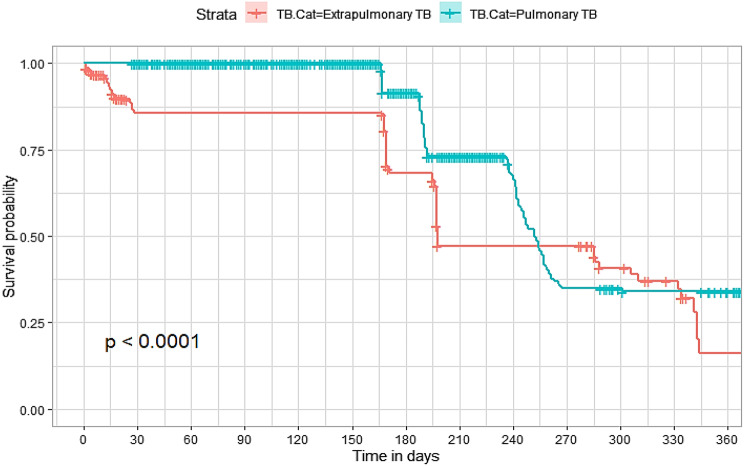
Survival curves for TB category for the study participants

**Fig. 8:**
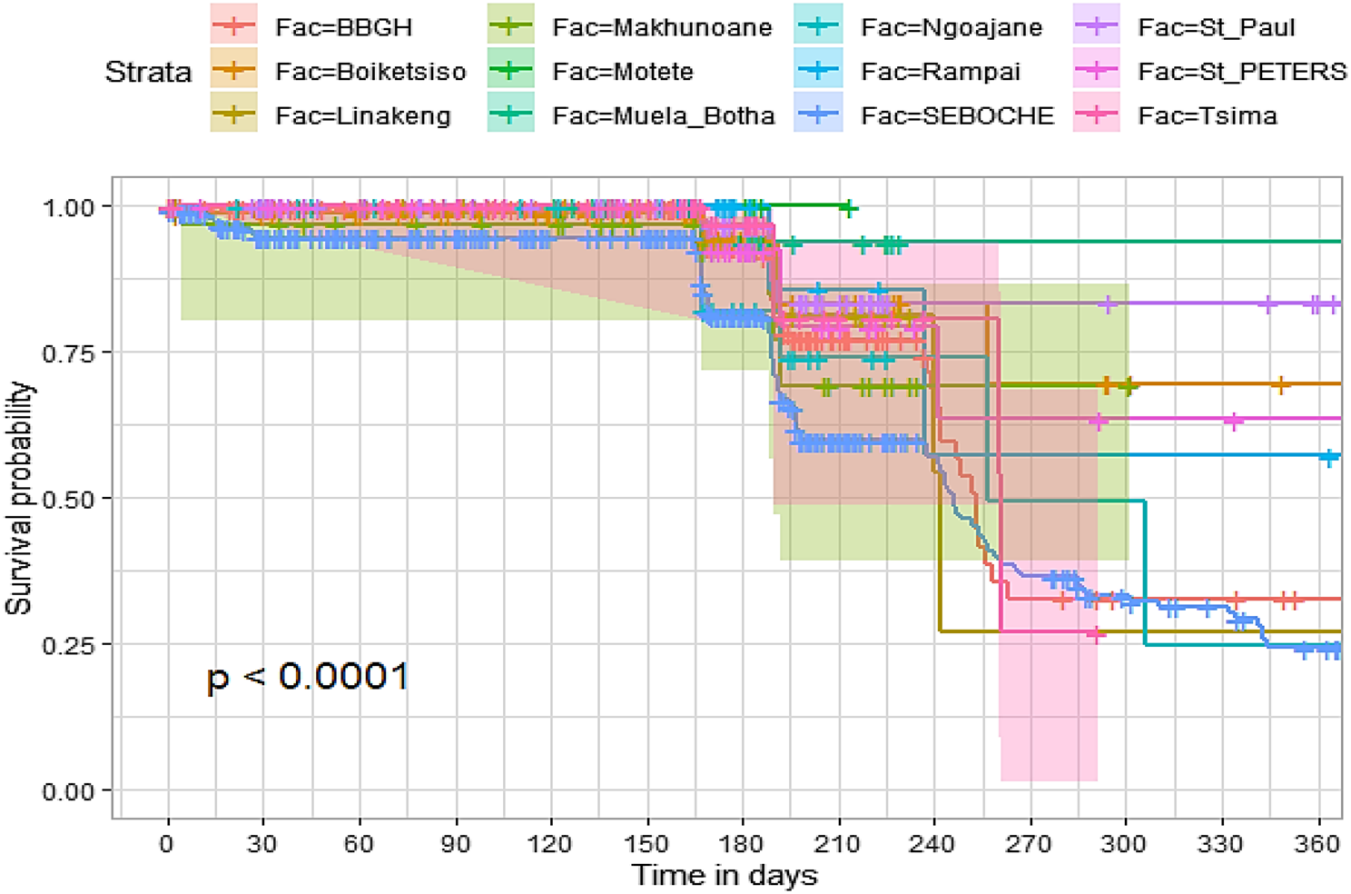
Survival curves for healthcare facility offering TB services

The survival distributions of time to death of the TB patients were estimated for each group using the Kaplan–Meier (KM) method to compare the survival curves of two or more groups. The Kaplan–Meier estimated survival curves in Figs. 1, 2, 3, 4, 5, 6, 7, 8, highlight the overall estimated survival function for each covariate. The overall estimated survival curve for the time to death of TB patients is shown in Fig. 1. In addition, the survival curves of TB patients were different for the different phase of treatment, treatment contact, category of TB, age category and catchment areas (facility) (Figs. 1, 2, 3, 4, 5, 6, 7, 8).

The observed differences in survival experiences in different patient groups was also assessed using the log-rank test. Survival time difference was significant at 5% significance level among phase of treatment, treatment contact, drug resistance, category of TB, age category and facility for TB patients (Table 2). The results complement the graphical presentation shown on the figures above.

**Table 2:** Chi-square estimate (${x^2}$) and *p*-value of log-rank tests for comparing the survival experience of TB patients from randomly selected hospitals in Lesotho

Covariate	*X*^*2*^	p
Sex	0.26	0.61
Treatment category	6.44	0.095
Age category	281.92	<0.001
Treatment contact group	24.62	<0.001
Employment status	5.04	0.025
Phase of treatment	125.34	<0.001
TB category	37.25	0.001
Facility	57.48	<0.001

## Model assessment and evaluation

To identify sets of covariates that have the potential influence to be included in the linear components of a multivariable model, literature review from similar studies was conducted. Given, fewer covariates that were abstracted from the database, we included all in the multivariable analysis. We performed multivariable survival analyses by assuming exponential, Weibull, lognormal, and Log logistic distributions for baseline hazard functions and varied the two frailty distributions for each one of them. Covariates with unambiguous estimates such as category of treatment and drug resistance were dropped from all the models.

Exponential, Weibull, Gompertz, lognormal and log logistic regression models without frailty were fitted first using INLA framework. Later, we varied the distributions of the frailty term to determine the best distribution for the frailty parameter being estimated using TB data from Butha Buthe district, Lesotho. We implemented Gamma and inverse-Gaussian distributions to capture the heterogeneity levels because there are the most common and widely used distributions cited in the literature [8, 16]. However, in this work, the distribution of the data was used to determine the appropriate frailty distribution (Table 3) supporting the observed data.

**Table 3:** Model assessment and evaluation

No	Distribution	WAIC Values
**a.Fixed Effect**		
	Weibull	−13.49
	**log logistic**	**−126.50**
	Exponential	40.95
	lognormal	−121.63
	Gompertz	43.19
**b.Gaussian distribution for frailty term**		
	Weibull	−69.40
	**Log logistic**	**−185.70**
	Exponential	−29.92
	Lognormal	−175.18
	Gompertz	−28.07
**c.Gamma distribution for the frailty term**		
	Weibull	−17428.47
	Exponential	1702436.92
	**log logistic**	**−25587.79**
	lognormal	−25576.55
	Gompertz	−25456.42

Table 3 shows the model distribution with related watanable akaike information that generalizes the WAIC for Gamma and Inverse-Gaussian shared frailty model. The model exponential is the poorest performing model in comparison to the other models with Gamma and Gaussian shared frailty terms. This is as shown by its highest WAIC value. The leading performing model is the Log logistic with or without the frailty term for both Gamma and Gaussian distribution, hence the strongest and decisive model is the log logistic model with a Gamma shared frailty distribution model. Thus, the concluding analysis and results interpretation were established on the logistic regression model with a Gamma frailty term.

We estimated five models that assume that TB patients from the same catchment area have uncorrelated survival risk. Furthermore, ten additional models were constructed to control for heterogeneity effect at the catchment level. Inclusion of the frailty term strategy enabled us to examine the effects of unobserved factor estimates of the known determinants for TB. Fixed effects results from the standard Weibull, Exponential, Lognormal, Gompertz and Log logistic models constructed to analyze the TB data are presented in Table 4.

**Table 4:** Fixed effect models with gamma frailty distribution

Variable	Weibull frailty			Loglogistic frailty			Exponential frailty			Lognormal frailty			Gomptez frailty		
	HR	2.50%	97,50%	HR	2.50%	97.50%	HR	2.50%	97.50%	HR	2.50%	97.50%	HR	2.50%	97.50%
**Sex**															
Female	Reference														
Male	1.33	0.30	4.72	0.79	0.18	3.79	1.34	0.30	5.81	0.61	0.09	3.79	1.29	0.23	6.54
**Age category**															
<20 Yrs	Reference														
20–59 Yrs	0.09	0.02	0.46*	12.01	1.59	95.00*	0.08	0.02	0.39*	5.11	0.98	73.43	0.05	0.02	0.45*
≥60 Yrs	3.25	0.98	18.40	0.17	0.04	0.74*	5.19	0.89	18.16	0.08	0.01	0.63*	3.76	0.87	22.01
**Occupation**															
Employed	Reference														
Mine Worker	0.37	0.21	0.78*	4.00	1.15	14.44*	0.31	0.09	0.77*	3.1	0.60	13.16	0.25	0.06	0.98*
Unemployed	0.67	0.09	3.81	2.35	0.45	23.01	0.43	0.08	2.73	5.43	0.67	73.24	0.61	0.07	6.01
**Treatment. contact**															
CHW	Reference														
Family member	1.53	0.29	11.06	0.39	0.07	3.07	1.53	0.30	11.02	0.41	0.02	3.12	0.99	0.16	5.71
Friend	0.81	0.08	6.06	1.84	0.17	19.13	0.89	0.09	8.11	2.51	0.09	69.57	0.68	0.07	7.33
Spouse	0.22	0.02	2.54	5.14	0.82	79.34	0.19	0.04	1.70	11.00	0.56	428,50	0.13	0.02	1.21
Unknown	0.52	0.06	2.22	2.67	0.28	19.57	0.47	0.09	3.24	3.56	0.24	74.00	0.29	0.03	2.17
**Employment Status**															
Employment															
Unemployed	0.69	0.14	3.55	1.29	0.34	11.97	0.89	0.11	4.23	1.67	0.34	17.23	0.72	0.08	7.49
**Phase of treatment**															
Phase 1															
Phase 2	0.27	0.09	0.79*	7.01	1.99	27.71*	0.35	0.09	0.79*	9.45	3.12	45.6*	0.33	0.08	1.34
**Treatment Category**															
CAT 1															
CAT 2	0.30	0.11	0.93*	2.55	0.88	10.71	0.31	0.10	0.90*	2.66	0.34	6.29	0.03	0.02	0.10*
CAT 3	1.17	0.15	7.89	1.20	0.14	11.30	1.44	0.26	8.38	0.75	0.03	24.31	0.69	0.09	6.17
CAT 4	-	-	-	-	-	-	-	-	-	-	-	-	-	-	-
TB Category															
**Extra Pulmonary**															
Pulmonary TB	1.12	0.37	3.30	1.58	0.39	5.71	0.94	0.35	3.41	4.11	0.94	23.76	1.43	0.41	4.13

## Determinants of TB mortality in Lesotho

Table 5 shows the hazard rates of TB mortality in twelve health facilities considered in the study with a Bayesian log logistic regression modeling approach via a Gamma shared frailty and presents estimates of fixed risk factors resulting from the models with the best fit. It is interesting to note that a male TB patient had reduced risk of dying relative to female patient, however this was not statistically significant (HR 0.59, Cl 0.08 - 3.85).

While being over 60 years, being contacted by community health worker and close relatives reduced the hazard of dying. Being over 60 years (HR 4.27, Cl 0.91–20.42) and being contacted by a friend (HR 0.77, Cl 0.08 -7.32) were associated with lower hazard of dying. Those who contacted close family relatives were not significantly different from those who did not (HR 1.68, Cl 0.28 - 9.95), even though the effect was found to be increased.

Interestingly, the effect of occupation on time of death was found to be increasing. Those who were employed as miners were 4.13 times more at risk of dying from TB (HR: 4.13, Cl 1.05–16.43). The effect was also positive for those who were unemployed but living in the same mining area (HR 1.18, Cl 0.25–12.9). This effect was however not statistically significant.

Analyzing the effect of employment status alone on mortality without stratifying for mining (miner and non-miner) masked the effect of occupation on death. The results showed a non-significant relationship (HR: 1.25, Cl: 0.64–2.45). The overall risk of death also reduced from 1.70 to 1.25. This finding shows that being in employment or not, did not significantly affect TB mortality for those living in the study area, however working as a miner increased the risk of dying, as reported above.

The effect of treatment category phase 2 (HR:6.98, Cl 1.78 - 27.69) increased the risk of dying relative to those in phase 1. While the effect of pulmonary TB (HR: 4.09, Cl 0.83 - 23.43) on mortality was 4.09 times higher than those who were diagnosed with extra pulmonary TB however, the observed effect was not statistically significant.

## Modeling heterogeneity across facility catchment areas

Results from survival mixed effect models fitted to control for unobserved effects (catchment region) are reported in Tables 5 and 6. Table 5 presents results for estimates from models with Gaussian distribution for the random shared parameter, while estimates in Table 6 are those from models that included the Gamma parameter as the distribution of choice for the random or frailty parameter.

Gamma parameter from log logistic distribution was found to be negligible. The heterogeneity explaining the unobserved effect is represented by the theta ($\theta$) parameter. The theta ($\theta$) is the parameter of the TB patient showing that patients from the same catchment area may have similar characteristics or share the same frailty but differ between or from facility catchment area to another facility catchment area. This means that the probability of TB mortality may be similar within facility catchment area but different between facility catchment areas due to some characteristics that were not or could not be measured. We observed a catchment specific and overall, statistically significant difference of the heterogeneity effect, implying significant difference for probability among TB patients from facility catchment to facility catchment.

Further, heterogeneity value associated with the frailty effect in the log logistic regression with Gaussian frailty model presented in Table 5 was 2.13 and can be as low as 0.4 and as high as 11.63. The study found statistically significant heterogeneity (variance structure) indicating that the risk of survival among TB patients differ because of unobserved effects shared by patients from the same facility catchment areas. The estimated frailty effect as shown in Table 5 with Gaussian distribution specifications show that a TB patient’s death in a specific catchment area increases the risk of the death for the TB patient by (exp ($\sqrt {2.13}$) =4.30) times, compared to the overall risk reported using traditional survival model.

**Table 5:** Random effect with gaussian distribution

Variable	Weibull frailty			Loglogistic frailty			Exponential frailty			Lognormal frailty			Gomptez frailty		
	HR	2.50%	97,50%	HR	2.50%	97.50%	HR	2.50%	97.50%	HR	2.50%	97.50%	HR	2.50%	97.50%
**Sex**															
Female	Reference														
Male	1.36	0.32	5.72	0.82	0.18	3.84	1.36	0.32	5.74	0.59	0.08	3.85	1.33	0.27	6.63
**Age category**															
<20 Yrs	Reference														
20–59 Yrs	0.07	0.01	0.42*	12.96	1.68	99.65*	0.07	0.01	0.41*	7.11	0.84	73.43	0.05	0.01	0.36*
≥60 Yrs	4.27	0.91	20.42	0.14	0.02	0.94*	4.25	0.89	20.46	0.08	0.01	0.73*	4.83	0.93	25.54
**Occupation**															
Employed	Reference														
Mine Worker	0.27	0.08	0.98*	4.13	1.05	16.43*	0.27	0.08	0.98*	2.8	0.58	15.17	0.22	0.05	0.91*
Unemployed	0.46	0.07	2.85	3.38	0.44	26.46	0.46	0.07	2.93	5.81	0.46	90.22	0.51	0.05	5.40
**Treatment. contact**															
CHW	Reference														
Family member	1.71	0.29	10.06	0.39	0.05	3.07	1.68	0.28	9.95	0.20	0.01	3.06	0.92	0.14	5.86
Friend	0.76	0.08	7.06	1.84	0.14	24.17	0.77	0.08	7.32	2.29	0.08	72.57	0.65	0.05	7.32
Spouse	0.18	0.02	1.58	7.16	0.59	90.79	0.18	0.02	1.67	13.14	0.54	425.30	0.11	0.01	1.19
Unknown	0.44	0.06	2.89	2.46	0.28	22.57	0.44	0.06	3.02	3.42	0.20	71.54	0.26	0.03	2.13
**Employment Status**															
Employment															
Unemployed	0.66	0.11	3.97	1.82	0.25	12.90	0.66	0.11	4.00	1.49	0.12	19.19	0.72	0.07	7.40
**Phase of treatment**															
Phase 1															
Phase 2	0.25	0,08	0.78*	6.98	1.78	27.69*	0.25	0.08	0.79*	10.00	2.12	58.6*	0.30	0.08	1.12
**Treatment Category**															
CAT 1															
CAT 2	0.28	0.09	0.83*	2.41	0.63	9.47	0.27	0.09	0.80*	1.41	0.29	6.26	0.02	0.01	0.09*
CAT 3	1.07	0.13	8.50	1.16	0.10	13.93	1.04	0.13	8.30	0.70	0.02	23.29	0.67	0.07	6.13
CAT 4	-	-	-	-	-	-	-	-	-	-	-	-	-	-	-
TB Category															
**Extra Pulmonary**															
Pulmonary TB	0.97	0.29	3.29	1.55	0.36	6.67	0.99	0.29	3.36	4.09	0.83	23.43	1.03	0.27	3.89
theta(frailty)	1.57	0.71	3.53	2.13	0.4	11.63	1.53	0.24	9.35	2.45	0.43	14.1	2.25	0.45	11.4

## Evidence of health facility catchment area heterogeneity

Table 6 presents result from Gamma parameterized model, which shows that the heterogeneity value was less than 0.001. This variance value was negligible even though it was statistically significant. However, the results presented show similar hazard ratios for risk factors evaluated with confidence intervals that are close for both frailty log logistic models with Gaussian and Gamma model. This shows that the impact of the frailty term on estimates did not change significantly and the significance of any of the parameter estimated remained unchanged when the frailty component was introduced to account for the heterogeneity effect. Similarly, when the frailty distribution changed from Gamma to Gaussian distribution, the size of the effect changed. However, the size of the effect of the determinants included as covariates in the model, remained same in the presence of the frailty parameter. It also becomes apparent that the credible interval from Gamma frailty models were consistently narrower compared to the one from Gaussian frailty modelling framework in a marginally manner.

**Table 6:** Random effect using gamma frailty distribution

Variable	Exponential frailty				Weibull frailty				Log logistic frailty			Lognormal frailty			Gomptz frailty		
	HR		2.50%	97.50%	HR		2.50%	97.50%	HR	2.50%	97.50%	HR	2.50%	97.50%	HR	2.50%	97.50%
**Sex**																	
Female																	
Male	1.36		0.32	5.72	0.82		0.18	3.84	1.36	0.32	5.74	0.59	0.08	3.85	1.33	0.27	6.63
**Age category**																	
<20 Yrs																	
20–59 Yrs	0.07		0.01	0.42*	12.96		1.68	99.65*	0.07	0.01	0.41*	7.11	0.84	73.43	0.05	0.01	0.36*
≥60 Yrs	4.27		0.91	20.42	0.14		0.02	0.94*	4.25	0.89	20.46	0.08	0.01	0.73*	4.83	0.93	25.54
**Occupation**																	
Employed																	
Mine Worker	0.27		0.08	0.98*	4.13		1.05	16.43*	0.27	0.08	0.98*	2.8	0.58	15.17	0.22	0.05	0.91*
Unemployed	0.46		0.07	2.85	3.38		0.44	26.46	0.46	0.07	2.93	5.81	0.46	90.22	0.51	0.05	5.40
**Treatment contact**																	
CHW																	
family member	1.71		0.29	10.06	0.39		0.05	3.07	1.68	0.28	9.95	0.20	0.01	3.06	0.92	0.14	5.86
Friend	0.76		0.08	7.06	1.84		0.14	24.17	0.77	0.08	7.32	2.29	0.08	72.57	0.65	0.05	7.32
Spouse	0.18		0.02	1.58	7.16		0.59	90.79	0.18	0.02	1.67	13.14	0,54	425.3	0.11	0.01	1.19
Unknown	0.44		0.06	2.89	2.46		0,28	22.57	0.44	0.06	3.02	3.42	0,2	71.54	0.26	0.03	2.13
**Employment Status**																	
Employment																	
Unemployed	0.66		0.11	3.97	1.82		0.25	12.9	0.66	0.11	4.00	1.49	0.12	19.19	0.72	0.07	7.40
**Phase of treatment**																	
Phase 1																	
Phase 2	0.25		0.08	0.78*	6.98		1.78	27.69*	0.25	0.08	0.79	10	2.12	58.6	0.30	0,08	1.12
**Treatment Category**																	
CAT 1																	
CAT 2	0.28		0.09	0.83*	2.41		0.63	9.47	0.27	0.09	0.80*	1.41	0.29	6.26	0.02	0.01	0.09*
CAT 3	1.07		0.13	8.50	1.16		0.10	13.93	1.04	0.13	8.30	0.70	0,02	23.29	0.67	0.07	6.13
CAT 4	-		-	-	-		-	-	-	-	-	-	-	-	-	-	-
**TB Category**																	
Extra Pulmonary																	
Pulmonary TB	0.97		0.29	3.29	1.55		0.36	6.67	0.99	0.29	3.36	4.09	0.83	23.43	1.03	0.27	3.89
theta(frailty)	<0.001				<0.001				<0.001			<0.001			<0.001		

The effect of frailty terms has been demonstrated by looking at the effect of exponential regression estimates for sex, occupation, and employment status on the risk of death of the T.B patient. Results from the standard model for these covariates showed no significant effect. However, the size of the effects changed after including the Gamma frailty term.

Equally, though the use of gamma and Gaussian produced similar estimates, conflicting estimates were also noted. Changing baseline likelihood from log logistic to exponential distribution affected the significance of the covariates. Inserting Gamma frailty term revealed significant higher risk of dying (HR: 1.36, Cl 0.32 - 5.72) relative to the standard model in which gender is not a significant determinant of TB mortality. Employment as a mine worker also changed the direction of significance when adjusted for the frailty parameter (HR:0.27, Cl:0.08–0.98). However, finding out that the effect size was quite small, was enlightening because the estimates from the model that fit the data the best (log logistic regression) suggested the opposite (See Tables 4, 5 and 6).

Drug resistant tuberculosis and the treatment category variables demonstrated persistent inconclusive risk values in all the models constructed and fitted. We dropped the two variables across all the 15 models fitted and were only reported in descriptive table. Considerable changes in the effects of frailty term on the selected covariates used in the Gaussian model when the distribution of the frailty parameter was changed to Gamma were also noted. For example, the effect of working as a miner on the risk of death became apparent as the credible interval became wider in the Gamma distributed frailty model compared to what was reported in the Gaussian distributed frailty model.

## Discussion

The central question of this study was to quantify the distribution of the TB data from Lesotho and identify risk factors associated with TB mortality, which go beyond individual factors, and extend to include other factors such as the catchment areas. These factors were assumed to be best captured by assuming frailty-varying processes. In doing so, we applied a novel Bayesian framework which permitted estimation of risk at individual, and catchment area (facility) level in a unified Bayesian framework.

The Kaplan–Meier survival curves show that the time to death of TB patients was different among age category, treatment contact group, employment status, phase of treatment, type of TB, and catchment areas (facility). However, clear differences were not observed among covariate such as gender, and treatment category. This result is consistent with another study which reported that age, HIV, chronic kidney disease, stroke, cancer, and chronic liver disease and cirrhosis were significant risk factors for death [25].

The log-rank tests also showed that there was a significant difference among the employment status. Similarly, the Kaplan–Meier survivor estimates for age category, treatment contact group, employment status, phase of treatment, type of TB, and catchment areas (facility) showed significant differences. These findings are consistent with those of one study done in Ethiopia [26], which found that the survival experience of patients in different categories of tuberculosis differed significantly.

Similarly, our study showed that mortality due to TB was indirectly associated with increasing age. Our study revealed that in the older TB patients; the mortality was significantly reduced. This finding contrasts with the findings reported in Tanzania [27] which found that advanced age ≥ 45 years increased the risk TB mortality. These contrasted results can be expected especially where data from other countries were analyzed using semi parametric approaches without necessarily testing for the proportionality of the hazard of mortality and ascertaining the distribution of the frailty parameter from the analyzed data. Nevertheless, this result was consistent with what was previously reported [28].

Our results showed that working as a miner elevates the risk of dying with TB. Similar findings have been observed by Dharmadhikari and collaborators [29]. In their study, it was noted that TB was a leading cause of death among mineworkers and former mineworkers, causing over 20% of deaths overall [29]. Evidence of increased TB related deaths, exceeding the proportion of deaths due to mining accidents in the mining population, were also commonly observed. Other studies have also noted increased risk of TB mortality among miner workers [30–32]. For instance, Lyatuu et al. reported that relative to non-miners living in the same communities, mining workers had twice the risk of mortality associated with TB [30]. Data from our study were also consistent with results reported elsewhere [33], which showed that mine activities continue to be associated with a large TB mortality burden despite major efforts to ensure the safety in mining communities. This may indicate that mining worker’s TB patients usually have lower survival probability than non-mining workers TB patients. This finding is very striking and may need to be investigated further using either prospective designs or any designs that can ensure proper attribution.

Further, according to the result obtained from the multivariable log logistic–Gamma shared frailty model, the risk of death was higher in phase 2 of treatment category of TB patients relative to phase 1 and was statistically associated with the time to death of TB patients during treatment. This finding is consistent with those of a study conducted in the southern region of Ethiopia [28].

The risk of death for extra pulmonary TB patients was significantly different from that of pulmonary TB patients. This result is consistent with that of the study that was conducted in Ethiopia [34] including other studies that found type of tuberculosis (pulmonary, and extra pulmonary tuberculosis) to be significant predictive factors of mortality of tuberculosis patients [35].

Interestingly, in our study, gender, was found not to be a statistically significant factor affecting the survival of TB patients. This result is consistent with the studies that were conducted in Uganda and Brazil [36, 37] but contradicts the findings of other studies that were conducted in Finland and Singapore [38, 39] that have found male TB patients to be at higher risk of dying due to TB. The differences noted could be attributed to several reasons including use of fixed effect models without accounting for catchment area heterogeneity and arbitrary choice of distribution for the hazard function.

The multivariate analysis was conducted using the log logistic model with Gamma frailty approach. The log logistic Gamma frailty model was arrived at after a comparison was made with other parametric Gamma frailty models as illustrated earlier in the methods section. Discrimination and final selection of the best model (log logistic) for this dataset was made using Watanabe Akaike criterion. Using findings from both Gamma and Gaussian frailty models, heterogeneity affected significance of the determinants for TB mortality. The results showed a statistically significant community level effect on the risk of dying and demonstrated variations from catchment area to catchment area especially for results from Gaussian distribution. This approach is similar to those carried out by other researchers previously [12].

The measure from this work needs to be interpreted with caution due to limitation of the scope and few covariates considered as this shows limited data representation. Excluding covariates such as marital status, education, higher level factors such as cultural, perception, practice and spatial information might have limited the ability of the model to explain the risk factors influencing mortality acting at higher than individual level. Spatial and seasonal information is needed to capture temporality as well as proximate influence which might have helped to account for possible and temporality spatial effect. However, model complexity and instability in some instances affected the precision structure and effect size of the heterogeneity risk quantification. For instance, when gamma distribution was specified for the frailty term, the estimates heterogeneity become small. While when the distribution was changed to the Gaussian or log normal, the heterogeneity effect was estimated to be 2.13. This means that the estimated frailty indicates that a TB patient’s death in a particular region increases the risk of the death for the index TB patient by (exp ($\sqrt {2.13)}$ = 4.30 times relative to the overall risk of TB patient’s death. Also, the estimated frailty variance value of <0.001 estimated using log-log gamma frailty distribution denotes that every TB death is associated with $\exp (\sqrt {0.0001} ) = 1.01005$ increased risk of the indexed TB patient dying relative to the average risk of death.

Furthermore, the lack of socioeconomic, TB resistance data, and spatial data may bias the study’s findings. Our modelling approach can be considered as an extension of the traditional survival model and can be classified as a mixed effect model. These types of models have a complex structure, which is easily exploited using the Bayesian approach. Despite the complexity of our approach the results obtained from our approach are consistent with what has been reported previously (9–12). In light of our findings, we propose the subsequent recommendations: (1) Facilitation of enhanced analytical foundations for the execution of effective TB interventions, standardization of TB Treatment protocols, and financial support to confront the challenges faced by miners; (2) mobilization of new resources and stakeholders, including national governments, development partners, the private sector, community organizations, research institutions, and ex-miners associations, to address the determinants of TB among mineworkers; and (3) a collaborative, innovative initiative involving government, community, development, and private sector partners focused on combating TB in the mining sector of Lesotho.

## Conclusion

The findings as presented show a further step toward an improved understanding of the risk of TB survival among the people of Lesotho. The existence of heterogeneity at the facility-level necessitates significant consideration of interventions aimed at enhancing TB treatment outcomes in Lesotho. This study has provided additional data augmenting the fact that information about individual patients and facility catchment areas is important in understanding TB mortality disparities, which can be further disentangled by using more advanced statistical methodologies such as survival mixed effect models (frailty). The Bayesian Gaussian frailty distribution specification did not exhibit any problem with the fit of the data. However, even though the Gaussian distributed frailty model exhibited a good fit to the data when modelled through the log logistic proportional regression with frailty, it had higher DIC and WAIC values. More research, including longitudinal studies that incorporate spatial and socioeconomic data, is required to validate application of frailty models.

## Data Availability

The datasets analyzed in this study are available from the corresponding author on reasonable request.

## References

[1] World Health Organization. Global tuberculosis report 2023. Available from: https://www.who.int/publications/i/item/9789240037021.

[2] World Health Organization. WHO global lists of high burden countries for tuberculosis (TB), TB/HIV and multidrug/rifampicin-resistant TB (MDR/RR-TB), 2021-2025. background document. World Health Organization. Available on https://iris.who.int/handle/10665/341980.

[3] Osório D, Munyangaju I, Nacarapa E, Nhangave A V, Ramos-Rincon JM. Predictors of unfavourable tuberculosis treatment outcome in Bilene district, Gaza Province, Mozambique: a retrospective analysis, 2016-2019. SAMJ: South Afr Med J. 2022;112(3):234–39. 35380527

[4] Woldemichael B, Darega J, Dida N, Tesfaye T. Treatment outcomes of tuberculosis patients and associated factors in Bale Zone, Southeast Ethiopia: a retrospective study. J Int Med Res. 2021;49(2):0300060520984916. 33528276 10.1177/0300060520984916 PMC7871063

[5] Birhan H, Derebe K, Muche S, Melese B. Statistical analysis on determinant factors associated with time to death of HIV/TB Co-infected patients under HAART at debre tabor referral hospital: an application of accelerated failure time-shared frailty models. Vol. 13. Auckland, NZ: HIV/AIDS; 2021. p. 775. 10.2147/HIV.S319745 PMC8298824 34305411

[6] Thompson SG, Sharp SJ. Explaining heterogeneity in meta-analysis: a comparison of methods. Stat Med. 1999;18(20):2693–708. 10521860 10.1002/(sici)1097-0258(19991030)18:20<2693::aid-sim235>3.0.co;2-v

[7] Wang CY, Wang N, Wang S. Regression analysis when covariates are regression parameters of a random effects model for observed longitudinal measurements. Biometrics. 2000;56(2):487–95. 10877308 10.1111/j.0006-341x.2000.00487.x

[8] Janssen P, Duchateau L. Frailty model. In: International encyclopedia of statistical science. Springer; 2011. p. 544–46.

[9] Van Oirbeek R, Lesaffre E. An application of Harrell’s C-index to PH frailty models. Stat Med. 2010;29(30):3160–71. 21170910 10.1002/sim.4058

[10] Mollel EW, Todd J, Mahande MJ, Msuya SE. Effect of tuberculosis infection on mortality of HIV-infected patients in Northern Tanzania. Trop Med Health. 2020;48(1):1–10. 32355448 10.1186/s41182-020-00212-z PMC7184680

[11] Jabir YN, Aniley TT, Bacha RH, Debusho LK, Chikako TU, Hagan JE Jr, et al. Time to death and associated factors among tuberculosis patients in Southwest Ethiopia: application of shared frailty model. Diseases. 2022;10(3):51. 35997356 10.3390/diseases10030051 PMC9397083

[12] Woya AA, Tekile AK, Basha GW. Spatial frailty survival model for multidrug-resistant tuberculosis mortality in Amhara Region, Ethiopia. Tuberc Res Treat. 2019. 10.1155/2019/8742363 PMC6332868 30693105

[13] Clayton DG. A Monte Carlo method for Bayesian inference in frailty models. Biometrics. 1991, June;47(2):467–85. 1912256

[14] Hougaard P. A class of multivanate failure time distributions. Biometrika. 1986, Dec;73(3):671–78.

[15] Klein JP. Semiparametric estimation of random effects using the Cox model based on the EM algorithm. Biometrics. 1992;795–806. 1420842

[16] Bower H, Crowther MJ, Rutherford MJ, Andersson TML, Clements M, Liu XR, et al. Capturing simple and complex time-dependent effects using flexible parametric survival models: a simulation study. Commun Stat - Simul Computation. 2021, Nov;50(11):3777–93.

[17] Balan TA, Putter H. A tutorial on frailty models. 2020. 29(11):3424–54. 10.1177/0962280220921889 PMC7534210 32466712

[18] Gutierrez RG. Parametric frailty and shared frailty survival models. 2002. 1:22–44.

[19] Mostafa AA. Fitting the hazard ratio for cluster survival data with frailty effect. 2012. 2(6):5781–93.

[20] Martino S, Akerkar R, Rue H. Approximate Bayesian inference for survival models. Scand J Stat. 2011;38.

[21] Alotaibi RM, Rezk HR, Guure C. Bayesian frailty modeling of correlated survival data with application to under-five mortality. 2020. 1–24. 10.1186/s12889-020-09328-7 PMC7504601 32957954

[22] Wienke A. Frailty models in survival analysis. 2010, July;2017.

[23] Li D, Jones A, Banerjee S, Engelhardt BE. Bayesian multi-group Gaussian process models for heterogeneous group-structured data. J Mach Learn Res. 2025;26(30):1–34. PMC12463451 41019101

[24] Kudryavtsev A, Shestakov O. The estimators of the bent, shape and scale parameters of the gamma-exponential distribution and their asymptotic normality. Mathematics. 2022;10(4):619.

[25] Lo HY, Suo J, Chang HJ, Yang SL, Chou P. Risk factors associated with death in a 12-month cohort analysis of tuberculosis patients: 12-month follow-up after registration. Asia-Pac J Public Health. 2015, Mar;27(2):NP758–68. 22199154 10.1177/1010539511429591

[26] Tolosie K, Sharma MK. Application of cox proportional hazards model in case of tuberculosis patients in selected Addis Ababa health centres, Ethiopia. Tuberc Res Treat. 2014. 2014;536976. 10.1155/2014/536976 PMC3913475 24523962

[27] Bukundi EM, Mhimbira F, Kishimba R, Kondo Z, Moshiro C. Mortality, and associated factors among adult patients on tuberculosis treatment in Tanzania: a retrospective cohort study. J Clin Tuberc Other Mycobact Dis. 2021, Aug;24:100263. 34355068 10.1016/j.jctube.2021.100263 PMC8322306

[28] Ballayira Y, Yanogo PK, Konaté B, Diallo F, Sawadogo B, Antara S, et al. Time and risk factors for death among smear-positive pulmonary tuberculosis patients in the health district of commune VI of Bamako, Mali, 2016. BMC Public Health. 2021, May;21(1):942. 34006238 10.1186/s12889-021-10986-4 PMC8132441

[29] Dharmadhikari A, Smith J, Nardell E, Churchyard G, Keshavjee S. Aspiring to zero tuberculosis deaths among southern Africa’s miners: Is there a way forward? Int J Health Serv: Plann, Adm, Evaluation. 2013;43(4):651–64. 10.2190/HS.43.4.d 24397232

[30] Id IL, Id MSW, Id GL, Id AF, Dietler D. Plos global public health estimating the mortality burden of large-scale mining projects - evidence from a prospective mortality surveillance study in Tanzania. 2021. 1–14. 10.1371/journal.pgph.0000008 PMC10021452 36962075

[31] Howlett P, Said B, Mwanga E, Mbuya A, Nota M, Kon OM, et al. Confronting the growing epidemic of silicosis and tuberculosis among small-scale miners. Lancet Public Health. 2025, Feb, 25;S2468-2667(25)00014-3. 10.1016/S2468-2667(25)00014-3 40020695

[32] Ndlovu N, Musenge E, Park SK, Girdler-Brown B, Richards G, Murray J. Four decades of pulmonary tuberculosis in deceased South African miners: trends and determinants. Occup Environ Med. 2018;75(11):767–75. 29934377 10.1136/oemed-2017-104806

[33] Selvaraju S, Thiruvengadam K, Watson B, Thirumalai N, Malaisamy M, Vedachalam C, et al. Long-term survival of treated tuberculosis patients in comparison to a General population in South India: a matched cohort study. Int J Infect Dis: IJID: off Publ Int Soc Infect Dis. 2021, Sept;110:385–93. 10.1016/j.ijid.2021.07.067 34333118

[34] Demissie M, Kebede D. Defaulting from tuberculosis treatment at the Addis Abeba tuberculosis centre and factors associated with it. Ethiopian Med J. 1994, Apr;32(2):97–106. 8033883

[35] Fløe A, Hilberg O, Wejse C, Ibsen R, Løkke A. Comorbidities, mortality and causes of death among patients with tuberculosis in Denmark 1998-2010. A nationwide, register-based case-control study. Thorax. 2018, Jan;73(1):70–77. 28778918 10.1136/thoraxjnl-2016-209240

[36] Baluku JB, Mukasa D, Bongomin F, Stadelmann A, Nuwagira E, Haller S, et al. Gender differences among patients with drug resistant tuberculosis and HIV co ‑ infection in Uganda: a countrywide retrospective cohort study. BMC Infect Dis. 2021;1–12. 10.1186/s12879-021-06801-5 PMC8542192 34689736

[37] de S VPV, Paiva NS, Villela DAM, Bastos LS, de Souza Bierrenbach Al, Basta PC. Factors associated with death in patients with tuberculosis in Brazil: competing risks analysis. PLoS ONE. 2020;15(10):e0240090. 33031403 10.1371/journal.pone.0240090 PMC7544107

[38] Vasankari T, Holmström P, Ollgren J, Liippo K, Kokki M, Ruutu P. Risk factors for poor tuberculosis treatment outcome in Finland: a cohort study. BMC Public Health. 2007;7(1):291. 17935630 10.1186/1471-2458-7-291 PMC2099439

[39] Low S, Ang LW, Cutter J, James L, Chee CBE, Wang YT, et al. Mortality among tuberculosis patients on treatment in Singapore. Int J Tuberc Lung Disease: off J Int Union Against Tuberc Lung Disease. 2009, Mar;13(3):328–34. 19275792

